# Understanding cooperativity of microRNAs via microRNA association networks

**DOI:** 10.1186/1471-2164-14-S5-S17

**Published:** 2013-10-16

**Authors:** Young-Ji Na, Ju Han Kim

**Affiliations:** 1 Seoul National University Biomedical Informatics (SNUBI), Seoul National University College of Medicine, Seoul 110-799, Korea; 2 Division of Biomedical Informatics, Seoul National University College of Medicine, Seoul 110-799, Korea

**Keywords:** association network, cooperativity, co-transcriptomic expression profile, microRNA, module

## Abstract

**Background:**

MicroRNAs (miRNAs) are key components in post-transcriptional gene regulation in multicellular organisms. As they control cooperatively a large number of their target genes, they affect the complexity of gene regulation. One of the challenges to understand miRNA-mediated regulation is to identify co-regulating miRNAs that simultaneously regulate their target genes in a network perspective.

**Results:**

We created miRNA association network by using miRNAs sharing target genes based on sequence complementarity and co-expression patterns of miRNA-target pairs. The degree of association between miRNAs can be assessed by the level of concordance between targets of miRNAs. Cooperatively regulating miRNAs have been identified by network topology-based approach. Cooperativity of miRNAs is evaluated by their shared transcription factors and functional coherence of target genes. Pathway enrichment analysis of target genes in the cooperatively regulating miRNAs revealed the mutually exclusive functional landscape of miRNA cooperativity. In addition, we found that one miRNA in the miRNA association network could be involved in many cooperatively regulating miRNAs in a condition-specific and combinatorial manner. Sequence and structural similarity analysis within miRNA association network showed that pre-miRNA secondary structure may be involved in the expression of mature miRNA's function.

**Conclusions:**

On the system level, we identified cooperatively regulating miRNAs in the miRNA association network. We showed that the secondary structures of pre-miRNAs in cooperatively regulating miRNAs are highly similar. This study demonstrates the potential importance of the secondary structures of pre-miRNAs in both cooperativity and specificity of target genes.

## Background

MicroRNAs (miRNAs) play crucial regulatory roles in repressing mRNA translation or mediating mRNA degradation by targeting mRNAs in a sequence-specific manner [1]. MiRNA-mediated regulation has been found to encompass a wide spectrum of biological processes ranging from growth and development to oncogenesis [2-5]. In general, one miRNA can target more than one gene (*i.e.*, multiplicity), and one gene can be controlled by more than one miRNA (*i.e.*, cooperativity) [6]. Cooperative binding of one or several distinct miRNAs to a single target gene has been shown to be important for the functionality of miRNA-mediated gene regulation [6,7]. For example, genetic evidences in previous research suggest that the lin-28 gene is cooperatively regulated by the lin-4 miRNA and another unidentified miRNA [8]. Krek *et al. *[9] also presented that miR-375, miR-124 and let-7b jointly regulate *Mtpn*, providing evidence to support coordinate control of miRNAs in mammals. Studying the cooperativity of miRNAs is a substantial step for understanding the contribution of co-regulating miRNAs towards a complex interplay between miRNAs.

Recently several studies have attempted to develop methods to understand miRNA cooperativity. Boross *et al. *computed the correlations between the gene silencing scores of individual miRNAs [10]. Xu *et al. *also developed a computational method to identify significant synergistic miRNA pairs via functional co-regulating miRNAs that they jointly regulate [11]. Most of these studies however did not actually considered co-expression profiles of mRNAs and miRNAs. Considering that most miRNAs exert their functions through interactions with other miRNAs, an understanding of a miRNA network context using both co-expression pattern and the sequence complementarity between miRNAs and mRNAs is essential to discover the cooperative regulation of miRNAs.

In this paper, we propose a computational method to construct a miRNA association network (MRAN) by integrating multiple genomic data sources including miRNA-mRNA binding information and miRNA-mRNA co-expression profiles. While sequence complementarity-based miRNA-mRNA target relationships serve as a static set, dynamic expression profiles of miRNAs and mRNAs are used to identify those subsets that are concurrently active. We evaluate the miRNAs modules determined to be significantly cooperative by assessing the functional coherence of target genes co-regulated by the miRNA modules. Then, we apply graph theoretical methods to characterize MRAN and analyse whether miRNAs belonging to the network are associated with each other in a condition-dependent manner or not. Finally we report that co-regulating miRNAs tend to have structural similarities.

## Results

### Construction and characterization of miRNA association networks

**Figure 1:**
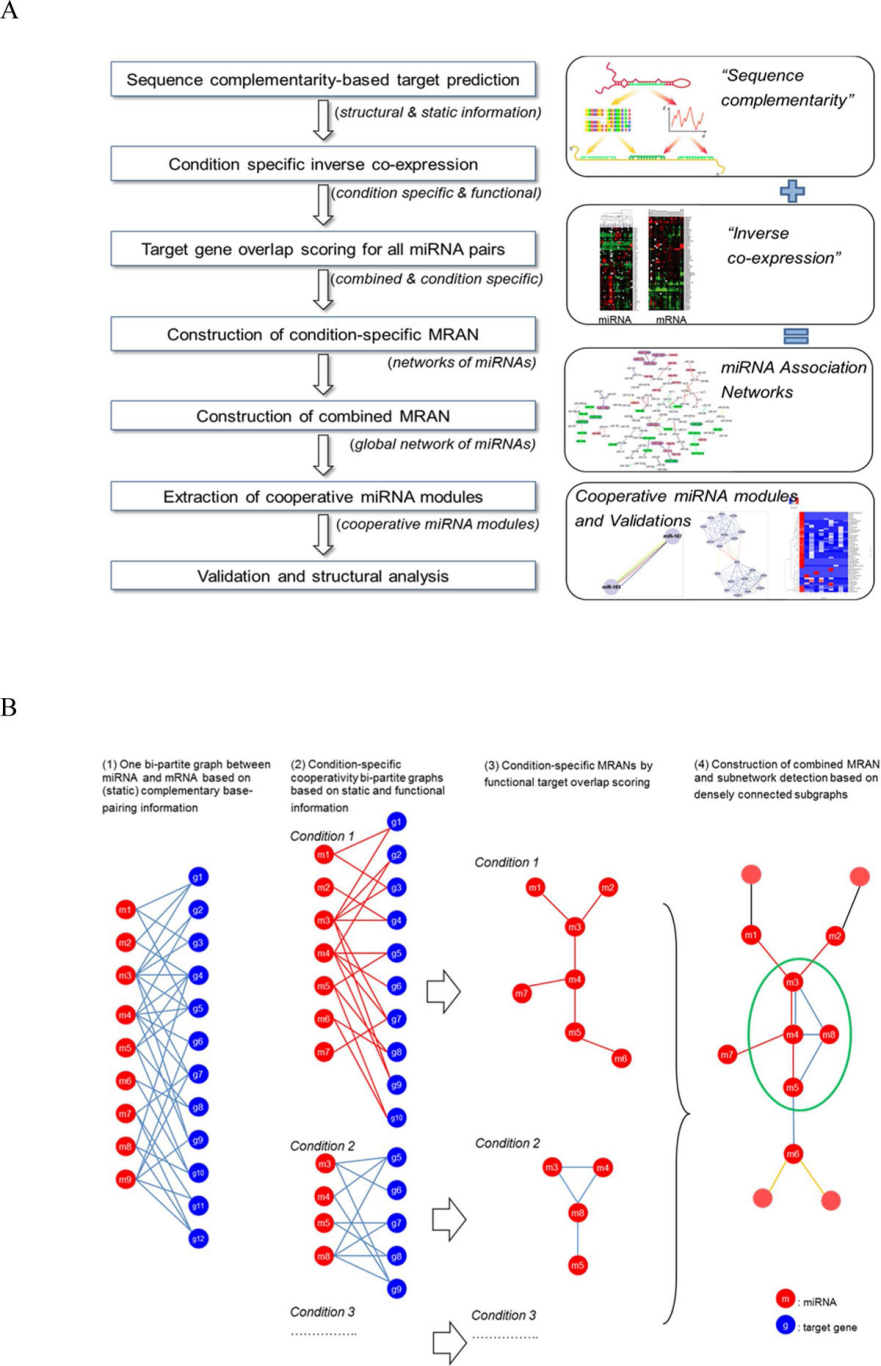
**Overview of identifying CMMs in MRAN**. (A) The conceptual diagram shows the stepwise process by which our methodology identifies cooperativity of miRNAs. The problem statement is outlined in the right column of the figure. (B) Schematic view of our approach to create miRNA association network and then to identify cooperatively regulating miRNAs in the miRNA association network.

**Figure 2:**
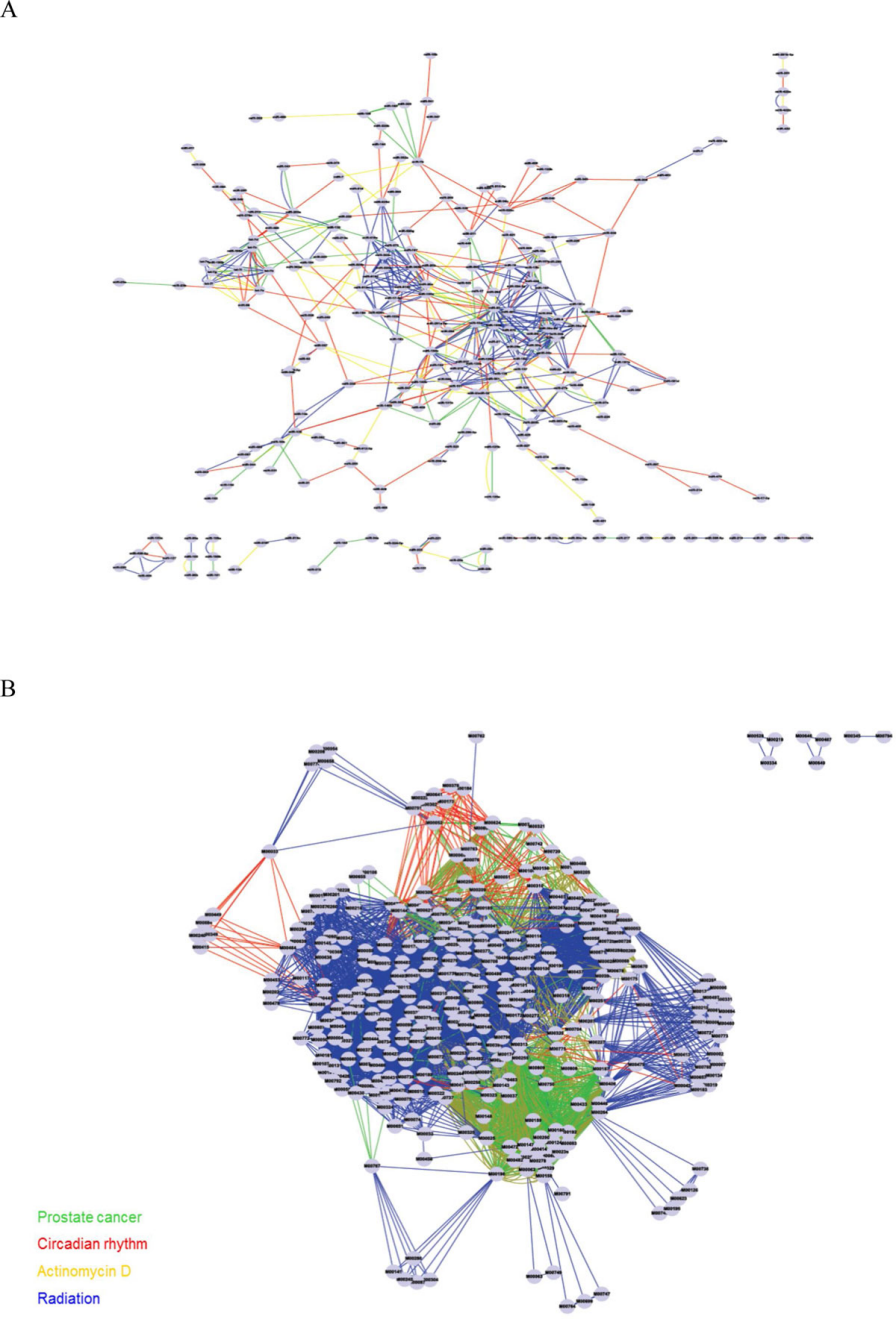
**Global MRAN and co-occurrence graph of regulators on MRAN**. (A) Global MRAN. Nodes of the network represent miRNAs, and edges represent condition-specific cooperativity between a miRNA pair. (B) Co-occurrence graph of regulators on MRAN. In the co-occurrence graph of regulators on MRAN, nodes represent transcription factors (TFs), and edges statistical significance of co-occurrences. The colors of edges indicate different conditions (red: circadian rhythm, blue: radiation treatment, yellow: Actinomycin D treatment, green: prostate adenocarcinomas).

Table 1 lists the four datasets used in the present study to create MRANs following the steps illustrated in Figure 1. Table 2 shows the distribution of miRNAs and mRNAs showing inverse expression pattern in each condition. Figure 2(A) shows the global MRAN created by superimposing four conditions specific MRANs (see Figure 1) consists of 241 miRNAs and 559 connections. In MRAN, a node corresponds to each miRNA that has the significant inverse expression pattern with its targets under each experimental condition (Pearson's correlation coefficient r < 0), and edges represent target overlap score p value < 0.05. MiRNAs were clustered into distinct groups.

**Table 1:** Co-transcriptomic expression datasets

Experiment	Sample	mRNA expression profile	miRNA expression profile	Reference
Circadian rhythm	Mouse liver	Illumina Mouse-6 Expression BeadChip	Ambion mirVana™ miR Bioarray V2	Na et al., 2009 [49]
ActinomycinD treatment	Mouse brain neuroblast N2a cells	Affymetrix Mouse Gene 1.0 ST Array	Ambion mirVana™ miR Bioarray V2	Unpublished data
Prostate cancer	Human prostate adenocarcinomas	Affymetrix Human Gene 1.0 ST Array	OSU-CCC MicroRNA Microarray	Prueitt et al., 2008 [37]
Radiation treatment	Human lung cancer H460 and H1229 cells	Affymetrix Human Gene 1.0 ST Array	Ambion mirVana™ miR Bioarray V2	Lee et al., 2008 [36]

**Table 2:** Distribution of miRNAs and mRNAs with inverse expression patterns

Category	Circadian rhythm	ActinomycinD	Prostate cancer	Radiation
No. of miRNAs	266	275	157	302
No. of genes	638	2446	1268	4354
No. of miRNA-gene pairs	1104	5095	2673	20522

**Figure 3:**
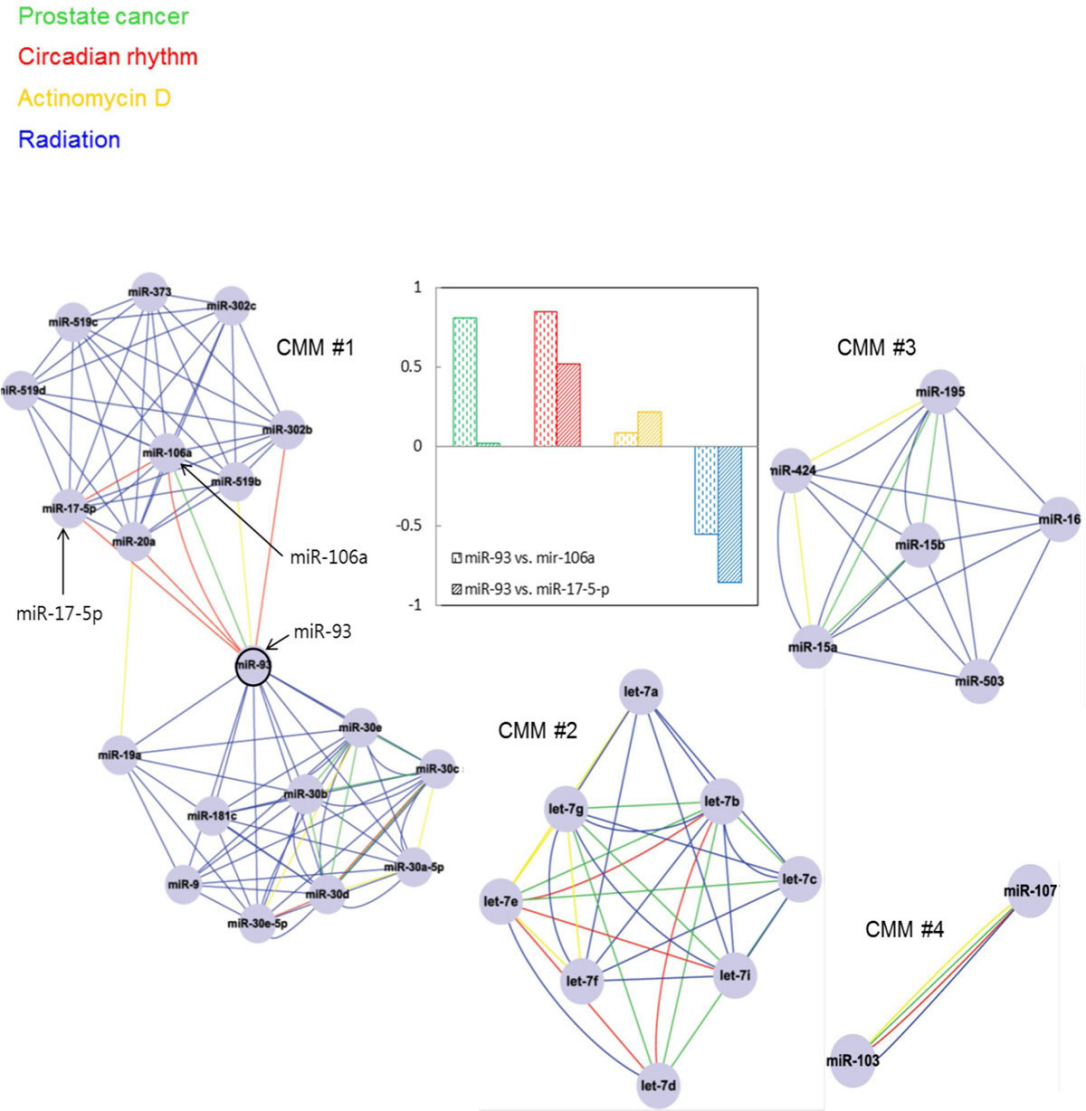
**One miRNA participating in multiple condition specificity**. The four representative CMMs identified with MCODE. The layout of this network was produced with Cytoscape [48]. Expression profiles of miR-93 and its partner miRNAs according to different conditions show correlation patterns.

Molecular COmplex DEtection algorithm (MCODE) [12] revealed 12 Cooperative MiRNA Modules (CMM) (Table 3). The numbers of miRNAs and connections of the CMMs range from 2 to 19 and 2 to 96, respectively (Table 3). CMM 2 and 4 consist of let-7 family members and miR-103/107 family, respectively. Table 3 exhibits miRNA family members included in CMMs and Figure 3 shows their condition-specific cooperativity. The first known human miRNA *let-7 *and its family members are highly conserved across species in sequence and function. Misregulation of *let-7 *leads to a less differentiated cellular state and the development of cell-based diseases such as cancer. The role of miR-103 and miR-107 in regulation of CDK5R1 expression and in cellular migration and neural development is well documented [13].

**Table 3:** Cooperative miRNA modules extracted from global MRAN

Subnetwork	No. of miRNAs	No. of links	No. of targets	miRNA family list
1	19	96	1536	miR-30a-5p, miR-30b, miR-30c, miR-30d, miR-30e, miR-30e-5p
				miR-106a, miR-20a, miR-519d, miR-17-5p, miR-93
				miR-302b, miR-302c, miR-373
				miR-519b, miR-519c
				miR-181c
				miR-19a
				miR-9

2	8	32	361	let-7a, let-7b, let-7c, let-7d, let-7e, let-7f, let-7g, let-7i

3	6	20	403	miR-15a, miR-15b, miR-16, miR-195, miR-424
				miR-503

4	2	4	100	miR-107, miR-103

5	4	7	552	miR-130a, miR-130b, miR-301
				miR-106b

6	3	5	214	miR-132, miR-212
				miR-194

7	3	5	349	miR-29a, miR-29b, miR-29c

8	4	6	105	miR-185 miR-198 miR-326 miR-7b

9	5	7	653	miR-26a, miR-26b

				miR-181a, miR-181d
				miR-101

10	3	3	10	miR-302d, miR-520b
				miR-349

11	2	2	33	miR-422a, miR-422b

12	3	3	132	miR-10a, miR-10b
				miR-339

Transcription factors are thought to regulate the transcription of miRNAs in a pol II dependent manner similar to that of protein-coding genes; that is, by binding to conventional transcription factor binding site sequences (TFBS) located in or near promoter regions that lie upstream of the miRNAs [14,15]. We examined the promoter regions of miRNAs for the presence of regulatory motifs. We determined over-represented motifs and found 285 significantly over-represented TFBS pairs (1 in prostate cancer, 241 in radiation, 6 in Actinomycin-D treatment and 37 in circadian rhythm) among the 6790 pairs (*i.e*., 766 in prostate cancer, 5105 in radiation, 744 in Actinomycin-D treatment, and 175 in circadian rhythm) using hypergeometric distribution (Bonferroni corrected *p*-value < 1.0e-05) (Figure 2(B)).

### Mutually exclusive functional landscape of miRNA cooperativity

**Figure 4:**
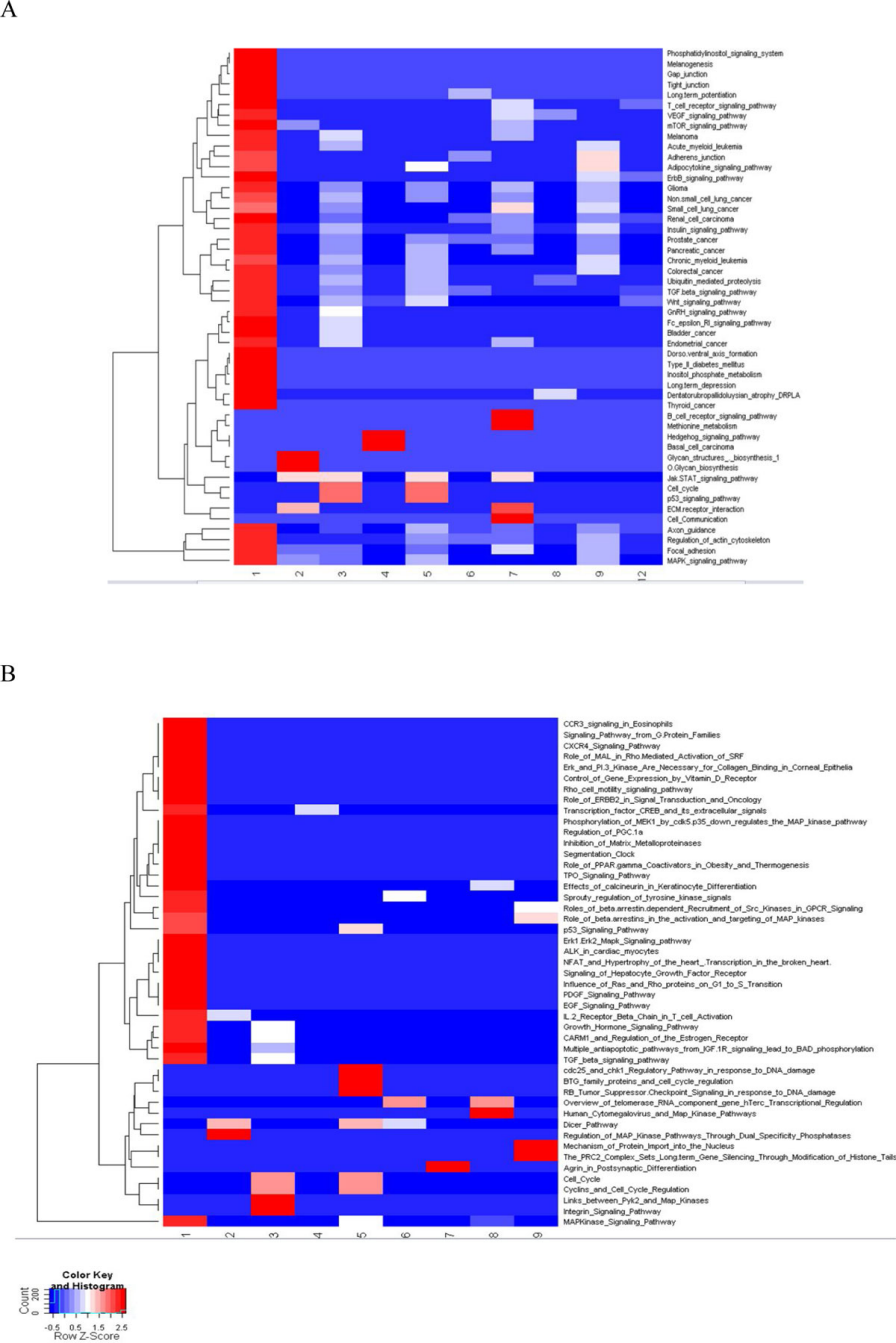
**Heatmap of over-represented biological pathways of target genes of CMM**. Color gradient represents statistical significance as the -log10 (p-value) in the hypergeometric test for enrichment analysis using (A) KEGG pathway and (B) BioCarta pathway. Red indicates significant associations while blue indicates insignificant associations.

The biology of miRNA function will be dictated by the mRNA transcripts targeted by specific miRNAs [16]. Functional enrichment analysis of miRNA's target transcripts hence can be used as a proxy for evaluation of the functional coherence of CMMs. We performed functional enrichment analysis by using all KEGG and BioCarta pathways for each CMM. Heat maps in Figure 4(A) and 4(B) exhibit-log_10 _*p*-values obtained by hypergeometric tests between (horizontal axis) CMMs' target transcripts and (vertical axis) KEGG and BioCarta pathways. All pathways mapped to at least one CMM and all modules mapped to at least one pathway appear in the Heat maps. Those modules and pathways that have no mapping at all are omitted from each heat map. Ten (*i.e*., CMMs 1~9 and 12) and nine (*i.e*., CMMs 1~9) CMMs have at least one KEGG and BioCarta pathway mappings, respectively.

CMM 1 has many significantly enriched pathways both in KEGG and BioCarta and other CMMs show small numbers of differently enriched pathways. One noteworthy pattern in both heat maps is the finding that functionally enriched pathways (in red) show little overlap between CMMs (Figure 4). It seems evident from the heat maps that CMMs extracted by mining the global MRAN exhibit functional landscape of mutually exclusive CMMs.

### Properties of the condition-dependent miRNA association network

One miRNA may be involved in many CMMs in a condition-specific and combinatorial manner. Figure 3 illustrates CMMs 1 to 4 from Table 3. Expression profiles of miR-93 and miR-106a show very high correlation coefficient (>0.8) as well as high target overlap score (*i.e*., shared target genes and inverse co-expression patterns with their shared target transcript) both in prostate cancer and circadian rhythm. So do the expression profiles of miR-93 and miR-17-5p in circadian rhythm. It seems that the function of a miRNA in the context of cooperativity can better be defined by its interactions with other miRNAs (or 'the company it keeps') rather than by its individual characteristics. Microarray analysis by Volinia *et al. *for determining miRNA signatures in prostate cancer includes both miR-93 and miR-106a, which have well-characterized cancer associations [17]. Several lines of evidences suggest that miR-93 may have different partners in different conditions. miR-93 and miR-130b affect the proliferation and survival of HTLV-1-infected/transformed cells [18]. miR-93 and miR-98 are expressed at higher levels in small-cell than in non-small-cell lung cancer cell lines and immortalized human bronchial epithelial cells (HBEC) [19]. MiRNAs-93, 92, 21, 126 and 29a were significantly over-expressed in the serum from ovarian cancer patients compared to controls [20].

The three miRNA families in CMM 3 are in fact regarded as a larger family of miR-15/107 group. These miRNAs are involved in cell division, metabolism, stress response, and angiogenesis in vertebrate species and have been implicated in human cancers, cardiovascular disease and neurodegenerative disease, including Alzheimer's disease [21]. Membership in this group is defined based on sequence similarity near the mature miRNAs' 5' end: all include the sequence AGCAGC. While all vertebrates studied to date express miR-15a, miR-15b, miR-16, miR-103, and miR-107, mammals alone are known to express miR-195, miR-424, miR-497, miR-503, and miR-646.

### Sequence and structural similarities within miRNA association network

Recognition of only 6 ~ 7 nt base pair in the seed sequence of the miRNA are enough to induce functional inhibition of the target gene [22]. Although critical points for target recognition are the short sites that match the seed region of the miRNA, the possible role of the secondary structure of miRNA cannot be overlooked in post-transcriptional regulation of miRNA expression. If we assume that the distributions of the similarities obtained within CMMs, between CMMs, and among random miRNAs is a normal distribution, the appropriate test is Wilcoxon signed rank test. Table 4 shows the results from statistical comparison of the distributions of the similarities. As expected, seed sequences within CMMs were significantly more similar than those between CMMs. In contrast, mature miRNA sequence did not achieve statistical significance. Neither did precursor miRNA sequence.

**Table 4:** Sequence and structure similarities of cooperative miRNA modules

		Comparison between CMMs		CMMs vs. random miRNAs	
			
		*H. sapiens*	*M. musculus*	*H. sapiens*	*M. musculus*
Mature form	seed sequence	1.87E-01 *	2.59E-01 *	5.50E-02 *	1.18E-01 *
	mature sequence	3.67E-01	8.77E-01	8.97E-01	9.96E-01

Precursor form	Sequence	6.21E-01	4.59E-01	3.52E-01	1.35E-01
	secondary structure	3.19E-08 *	1.06E-03 *	< 2.2e-16*	< 2.2e-16*

*p < 0.005

Very interestingly, however, secondary structure of precursor miRNA showed statistically significant differences such that precursor structure comparison showed even much smaller *p*-values than seed sequence comparison, which is believed to be the primary regulatory element of miRNA. Although CMMs tend to include miRNA families as shown in Table 3, structural similarity of pre-miRNAs within CMM cannot be explained by miRNA families because many of them originate from different precursors. Moreover, a CMM tends to contain more than one miRNA families and structural similarity of pre-miRNA is even bigger than seed sequence similarity (Table 3). It is suggested that pre-miRNA secondary structure may be involved in the expression of mature miRNA's function.

### Evaluation

To evaluate the efficiency of our pipeline, we compared target gene enrichment scores of our miRNA modules with those of miRNA modules from previous study using enrichment scores provided by DAVID Gene Functional Classification [23].

The enrichment scores are intended to order the relative importance of the gene groups. A higher score indicates that the group members are involved in more important (enriched) roles. The enrichment score of each group is measured by the geometric mean of the EASE Scores (modified Fisher Exact) associated with the enriched annotation terms that belong to this gene group.

**Figure 5:**
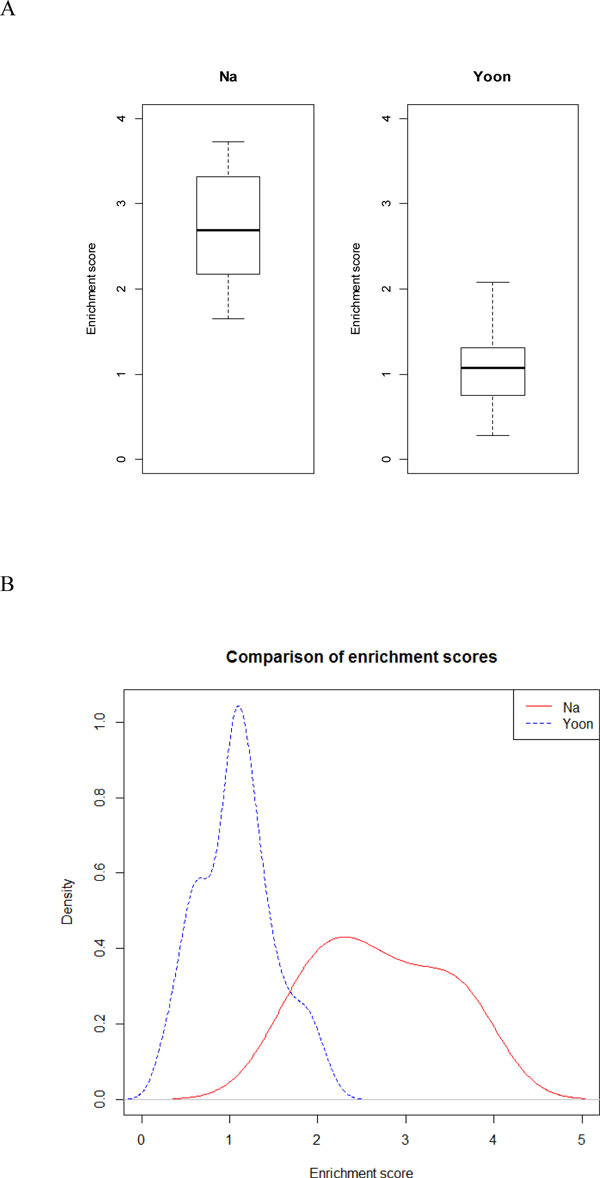
**Comparison of enrichment scores of target genes between this study and previous study**. We made (A) boxplot and (B) density plot in order to compare the distribution of enrichment scores of target genes between this study and previous study. These plots show that the enrichment scores of targets genes in the miRNA modules obtained from this study are statistically significantly higher than those from previous study.

We used Wilcoxon Rank Sum test to compare target gene enrichment scores between this study and previous study. A statistically significant difference in the target gene enrichment score distribution was observed (p-value = 1.495e-06). In addition, in order to compare the distribution of enrichment scores of target genes between this study and previous study, we made boxplot and density plot (Figure 5(A,B)). These plots show that the enrichment scores of targets genes in the miRNA modules obtained from this study are statistically significantly higher than those from previous study. In conclusion, we can demonstrate that our pipeline for finding miRNA modules is more efficient than previous method in terms of enrichment of target gene function.

## Discussion

MiRNAs can bind to one gene [24-26] and the target sites may overlap to some degree [27]. In many studies, individual effect of a miRNA may appear to be small but when they cooperate, the effect can be of significant proportions [28]. Sætrom *et al. *(2007) observed that lin-41 down-regulation in C. elegans requires cooperativity between three miRNA sites [29]. Mavrakis *et al. *(2011) showed that a small set of miRNAs are responsible for the cooperative suppression of several tumor-suppressor genes in T-cell acute lymphoblastic leukemia (T-ALL) [30]. Cooperativity therefore provides the mechanistic basis for reading out combinatorial expression patterns of miRNAs.

Network-based approach of the present study aims to investigate cooperative translational control of miRNAs. We constructed MRAN and showed that CMMs share their transcription factors in the association network. Transcription factors may bind directly to the pri-miR and/or pre-miR to regulate their processing [31]. TFBS within pre-miRs might serve specifically to regulate transcription of the primary miRNA gene transcript (pri-miR) itself as well as transcription of nearby downstream genes. It may be a crucial factor that co-regulating miRNAs act cooperatively to target genes.

It is demonstrated that individual miRNA may interact with different miRNA partners in a condition-specific manner. Some miRNAs may act as 'global facilitator miRNAs' that assist their target-specific partners in their functions. They may inhibit their target genes depending on conditions and partner miRNAs, enabling a post-transcriptional response that integrates multiple environmental signals and pathways.

MiRNA biosynthesis can no longer be viewed as one general pathway universal to all miRNAs. Many steps can be performed in multiple ways, omitted or replaced, and are affected by different mechanisms for individual miRNAs. Most importantly, these specific differences in miRNA processing suggest multiple opportunities for post-transcriptional regulation of miRNA expression [32]. We found that the secondary structures of pre-miRNAs in CMMs are highly similar. On the contrary, pre-miRNAs in CMMs are not similar at the sequence level. The secondary structures of pre-miRNAs have been reported to serve as a signature motif that is recognized by the nuclear export factor Exportin-5 in the biogenesis of miRNA [33]. Structure-function study raises the possibility of the involvement of pre-miRNA secondary structure into specific functions of targets via different miRNA biogenesis pathways. It has been further reported that the processing of pre-miRNAs is specifically regulated [34]. These differential processes potentially may have caused selection of distinctive structural features that are involved in discriminating regulatory interactions. Although the seed sequences of miRNAs in CMMs are mostly similar (or identical), there is no one-to-one mapping between CMMs and seed sequences. Instead, CMMs seem to respond to a small group of similar seeds that may be shifted from the usual position (residues 2-8) to, e.g. the positions 1-7 or 4-10.

Our results highlight the potential importance of the secondary structures of pre-miRNAs in both cooperativity and specificity of target genes. Vermeulen *et al. *identified the features of miRNA structure that affects Dicer specificity and efficiency showing that various attributes of the 3' end structure which play a primary role in determining the position of Dicer cleavage [35]. In addition, the functional regulatory networks of miRNAs can provide insights into the intricacies of miRNA processing. The systematic study of identifying co-regulating miRNAs showed that specifically regulated processing of pre-miRNAs may have caused selection of distinctive structural features that are involved in discriminating regulatory interactions. It lends further credibility to the hypothesis that structural subclasses could be associated with processing differences of the precursors.

## Conclusions

Cooperative signal integration on target genes of miRNAs is key features of the control of translation by miRNAs. Here, we constructed MRAN using CMMs to investigate miRNA cooperativity based on integration of multiple genomic data sources. The functional regulatory networks of miRNAs can provide insight into the intricacies of miRNA processing. Pre-miRNA secondary structure is suggested to be involved in mature miRNA function. In conclusion, the molecular dissection of miRNA modulation will help to unravel their functional comprehension and escalate one level towards their molecular decoding.

## Methods

### Overview of finding co-regulating miRNAs

Steps for identifying co-regulating miRNAs are presented in Figure 1. Co-regulating miRNAs correspond to a group of miRNAs silencing together target genes. Sequence complementarity-based computational prediction can consider static target relation only but not cooperative binding of miRNAs that may occur in a condition-specific manner. One needs to systematically investigate expression patterns between miRNAs and mRNAs across different conditions. We used miRNA-mRNA co-expression profiles to select condition-specific modules of cooperative miRNAs at the level of functional expression. Using miRNAs which share common targets, we constructed MRAN and extracted clusters of tightly co-regulating miRNAs from MRAN based on the network analytic approach.

### Datasets

We first used miRNA-mRNA co-transcriptomic datasets obtained from the same samples by ourselves in our previous studies (Table 1). Circadian rhythm dataset [12] consists of triplicated mRNA (Illumina Mouse-6 Expression BeadChip) and duplicated miRNA (Ambion mirVana™ miRNA Bioarray V2) expression profiles from 12 time points with four-hour interval across two complete circadian cycles, *i.e.*, 48 hours, resulting 36 and 24 hybridizations for mRNA and miRNA, respectively. One can download the dataset at Gene Expression Omnibus (GSE11516). Radiation dataset [36] consists of triplicated mRNA (Affymetrix Human Gene 1.0 ST Array) and duplicated miRNA (Ambion mirVana™ miRNA Bioarray V2) expression profiles from 6 time points, resulting 18 and 12 hybridizations for mRNA and miRNA, respectively. Two lung cancer cell lines, H460 and H1299 were irradiated at 2 Gy, and harvested after 0, 2, 4, 8, 12 and 24 hours to examine the expressions. Actinomycin D dataset (unpublished data) is created to explore the bio-molecular mechanism of miRNA decay. It consists of triplicated mRNA (Affymetrix Mouse Gene 1.0 ST Array) and duplicated miRNA (Ambion mirVana™ miRNA Bioarray V2) expression profiles from 7 time points, resulting 21 and 14 hybridizations for mRNA and miRNA, respectively. N2a mouse neuroblastoma cells were treated with Actinomycin D to block transcription at 0, 1, 2, 4, 8, 12 and 24 h time points. We also included external dataset from 57 prostate adenocarcinomas (GSE7055) [37] (Table 1).

Normalizations of the miRNA and mRNA expression profiles were performed separately. For datasets using Affymetrix GeneChips, we used RMA (Robust Multi-Array) normalization method [38]. For datasets using Ambion miRNA chips and OSU-CCC chips, we used vsn transformation after background subtraction [39] and applied quantile normalization method [40]. For datasets using Illumina chips, we used chip-wise method using the rank invariant algorithm [41].

### Target overlap score and MRAN construction

MRAN is defined as a combined network of cooperative miRNAs sharing condition-specific target genes. A node in MRAN corresponds to a miRNA and an edge corresponds to a condition-specific cooperativity between a miRNA pair. Condition specific cooperativity is defined as condition specific target sharing and determined by (static) significant sequence complementarity by computational target prediction algorithms provided by TargetScan and (functional) significant inverse expression pattern at a specific experimental condition (by Pearson's correlation coefficient). We used the Pearson's correlation coefficient (PCC) for measuring similarity/dissimilarity between expression patterns of miRNAs and genes. The PCC of a pair of genes commonly returns a real value in [+1, -1]. PCC(x, y) > 0 represents that x and y are positively correlated with the degree of correlation. On the other hand, PCC(x, y) < 0 represents that x and y are negatively correlated with a value |PCC(x, y)|. A positive value of the PCC indicates that miRNAs and genes are co-expressed and a negative value of the PCC indicates that opposite expression pattern exists between them. Considering the conservation of miRNA-target relationship, we retrieved and applied 50,339 and 50,349 human and mouse miRNA-target pairs for the 162 and 7,927 conserved miRNAs and mRNAs by using TargetScan target prediction database (release 4.2) [42].

Target overlap score between a pair of miRNAs is defined as Jaccard similarity coefficient, representing the fraction of shared condition specific targets. We define the target overlap score *t_ij _*between miRNAs *i *and *j*as follows:

$$t_{ij}=\left\{\begin{array}{cc} \frac{\left|Targets\left(i\right)\cap Targets\left(j\right)\right|}{\left|Targets\left(i\right)\cup Targets\left(j\right)\right|}, & i\neq j\\ 1, & i=j \end{array}\right)$$

where *Targets(i)* represents to the set of targets of miRNAs *i*. We measured the statistical significance of the Jaccard similarity coefficient by using the exact randomization tests [43]. The *n *× *n *condition specific target overlap matrix *T *= [*t_ij_*] is transformed into an *n *× *n *adjacency matrix *A *= [*a_ij_*], which encodes the connection strengths between pairs of nodes based on the probabilities related to the Jaccard similarity coefficient. Since the networks considered here are undirected, *A *is a symmetric matrix with non-negative entries.

Figure 1 outlines the steps of the present study. Figure 1(B) conceptualizes (1) the miRNA-mRNA target relationship as a directed bi-partite graph at the sequence level. (2) Many condition-specific directed bi-partite graphs are emerging by combining inversely co-expression patterns between miRNA and mRNA pairs. (3) By means of applying target overlap scoring, many condition-specific MRANs are obtained as undirected weighted graph of miRNAs. (4) Many condition-specific MRANs are merged into a combined multiple graph to form global MRAN (see Figure 2(A)). Now one can extract cooperative miRNA modules (CMMs) by applying subnetwork detection algorithms and then characterize and evaluate them in terms of biological relevance.

### Extracting cooperative miRNA modules

In the present study, a subnetwork detection algorithm, called MCODE [12], was applied to detect coherent groups in the global MRAN. The MCODE is a graph theoretic clustering algorithm specifically designed to find complexes by identifying densely connected subgraphs in networks. MCODE algorithm consists of three stages: vertex weighting, complex prediction and an optional post-processing step. The weighting of nodes is based on the core clustering coefficient. Once the weights are computed, the algorithm traverses the weighted graph in a greedy fashion to isolate densely connected regions. The post-processing step filters or adds nodes based on connectivity criteria. MCODE has a parameter that specifies the size of clusters returned. All MCODE parameters are applied with default values. Subnetworks are filtered if they do not contain at least a 2-core (graph of minimum degree 2). This approach allows us to assign one miRNA to multiple clusters, considering a biological principle that miRNAs can have multiple functions.

### Regulators of cooperative miRNAs

To investigate the modular nature of global MRAN, we used predicted binding sites for all Position Specific Scoring Matrices (PSSMs) from TRANSFAC version 8.3, as they are defined by the UCSC hg17 genome assembly, in the tfbsConsSites (http://genome.ucsc.edu/) and tfbsConsFactors. All RefSeq genes genomic locations were taken from hg17. In mouse, regulators of miRNAs were identified in the human genome in the regions orthologous to the mouse. For example, hsa-miR-101 in human and mmu-miR-101 in mouse are orthologous. To assess the statistical significance of the rate of co-occurrence of motif pairs, we used cumulative hypergeometric distribution to calculate the probability of obtaining the rate of co-occurrence, *C*, equal to or higher than the observed rate of co-occurrence, *c'*, by chance:

$$P\left(C\geq c^{\prime }\right)=\overset{min\left(m_{1},m_{2}\right)}{\underset{i=c^{\prime }}{\sum }}\frac{\left(\begin{array}{c} m_{1}\\ i \end{array}\right)\left(\begin{array}{c} N-m_{1}\\ m_{2}-i \end{array}\right)}{\left(\begin{array}{c} N\\ m_{2} \end{array}\right)}$$

where *m_1 _*and *m_2 _*denote the numbers of miRNAs having each of the two motifs, *N *denotes the total number of miRNAs in the genome, and *i *the summation index.

In the co-occurrence graph of regulators on MRAN, nodes represent transcription factors (TFs), and edges statistical significance of co-occurrences. Hypergeometric *p*-values were used as weights (see Figure 2(B)).

### Functional coherence analysis of target genes

Plausible characteristics of an extracted subnetwork as a CMM might be functional coherence, meaningfulness as well as distinctness from other modules. We performed functional enrichment analysis by using KEGG (Kyoto Encyclopedia of Genes and Genomes) [44] and BioCarta (http://www.biocarta.com) pathways to test their functional coherence and meaningfulness. KEGG pathways mainly include cellular processes related to metabolism and biosynthesis. Those on BioCarta cover a wider variety of cellular processes including a large number of signal transduction and immune signalling pathways as well as metabolic and biosynthetic pathways. Pathways with *p*-value < 0.05 as revealed by the hypergeometric test were considered statistically significant in the present study. To visualize the distinctness of the cooperative modules in terms of functional enrichment, we created heat maps of the significances (see Figure 4).

### Sequence/structure analysis of the co-regulating miRNAs

Structural similarity of the members of CMMs was evaluated. T-COFFEE (version 5.05) [45] was used to calculate pairwise sequence similarity between miRNAs. It uses information from a pre-compiled library of different pairwise alignments including both local and global alignments. RNAdistance program of the Vienna RNA package (Version 1.7.1) [46] was applied to calculate structural distances. Sequence-structure based clustering using the LocARNA-RNAclust pipeline [47] was applied to pre-miRNA sequences. LocARNA uses a complex RNA energy model for simultaneous folding and sequence/structure alignment of the RNAs [47]. The resulting alignment scores can be used to cluster RNAs according to their sequential and structural similarities.

## Competing interests

The authors declare that they have no competing interests.

## Authors' contributions

YJN designed the study, performed data analysis and wrote the paper. JHK supervised the research.

## Declarations

Publication of this article was funded by the Ministry of Education, Science and Technology, Korea; the Ministry of Health and Welfare, Korea and the Ministry of Science, ICT & Future Planning, Korea.

This article has been published as part of *BMC Genomics *Volume 14 Supplement 5, 2013: Twelfth International Conference on Bioinformatics (InCoB2013): Computational biology. The full contents of the supplement are available online at http://www.biomedcentral.com/bmcgenomics/supplements/14/S5.
